# Parallel Quantum Circuit in a Tunnel Junction

**DOI:** 10.1038/srep30198

**Published:** 2016-07-25

**Authors:** Omid Faizy Namarvar, Ghassen Dridi, Christian Joachim

**Affiliations:** 1 CEMES-CNRS, 29 rue J. Marvig, 31055 Toulouse Cedex, France; 2 WPI-MANA, National Institute for Material Sciences, 1-1 Namiki, Tsukuba, Ibaraki, Japan

## Abstract

Spectral analysis of 1 and 2-states per line quantum bus are normally sufficient to determine the effective *V*_*ab*_(*N*) electronic coupling between the emitter and receiver states through the bus as a function of the number *N* of parallel lines. When *V*_*ab*_(*N*) is difficult to determine, an Heisenberg-Rabi time dependent quantum exchange process must be triggered through the bus to capture the secular oscillation frequency Ω_*ab*_(*N*) between those states. Two different linear and 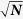 regimes are demonstrated for Ω_*ab*_(*N*) as a function of N. When the initial preparation is replaced by coupling of the quantum bus to semi-infinite electrodes, the resulting quantum transduction process is not faithfully following the Ω_*ab*_(*N*) variations. Because of the electronic transparency normalisation to unity and of the low pass filter character of this transduction, large Ω_*ab*_(*N*) cannot be captured by the tunnel junction. The broadly used concept of electrical contact between a metallic nanopad and a molecular device must be better described as a quantum transduction process. At small coupling and when *N* is small enough not to compensate for this small coupling, an *N*^2^ power law is preserved for Ω_*ab*_(*N*) and for *V*_*ab*_(*N*).

Connecting two identical *A* and *B* quantum systems using a quantum transfer line opens the possibility to transfer one electron from *A* to *B* because of the electronic coupling introduced between *A* and *B* by this line^1^,^2^. To increase the chance for this transfer, to speed it up or to minimize the energy required, *N* identical lines can be added in parallel forming a quantum bus between A and B^2^. In absence of mutual electronic coupling between the lines, the *V*_*ab*_(*N*) coupling between state |*ϕ*_*a*_〉 (the electron on *A*) and state |*ϕ*_*b*_〉 (the electron on *B*) must intuitively increase. Quantifying the *V*_*ab*_(*N*) power law increase as a function of *N* and measuring it experimentally are long standing problems^1^,^2^. A possible measure (i) is to perform a spectroscopy characterization of the *A*–*N*–*B* quantum system to follow how the degeneracy between |*ϕ*_*a*_〉 and |*ϕ*_*b*_〉 in absence of the bus is then lifted up by the progressive insertion of *N* lines in parallel between *A* and *B*. Measure (ii) protocol is to follow in real time the electron transfer process between *A* and *B* and to measure how *N* is changing the Ω_*ab*_(*N*) Heisenberg-Rabi secular oscillation frequency of this process before any relaxation (for example the electron being trapped on *A* (on *B*) or ejected from *A*–*N*–*B*). Measure (iii) protocol is to connect *A* and *B* to conductive nanopads *M*_*A*_ and *M*_*B*_ interacting electronically respectively with |*ϕ*_*a*_〉 and |*ϕ*_*b*_〉, to low bias voltage the corresponding *M*_*A*_-A-N-B-*M*_*B*_ junction and to follow the variations of the *I*(*N*) current intensity through this junction as a function of *N*.

Since the first electron transfer experiments through a molecular wire^3^, measure (i) had long been performed. More recently, it had been used for mesoscopic qubit systems^4^ and to measure the electronic coupling between 2 metallic nano-cubes stabilized together by a small number *N* of short molecular wires self-assembled in parallel^5^. But for large *V*_*ab*_(*N*), |*ϕ*_*a*_〉 and |*ϕ*_*b*_〉 are quite difficult to identify in the overall spectrum because they are both very diluted on the *A*–*N*–*B* electronic eigenstates.

Measure (ii) is depending on the technical possibility to follow in real time very fast phenomena since even for *V*_*ab*_(*N*) of the order of a few *μeV*, Ω_*ab*_(*N*) = 2*V*_*ab*_(*N*)/*ħ*^6^ can already reach the GHz regime^4^,^7^. In the case of quantum decoherence along the bus (for example |*ϕ*_*b*_〉 not fully reconstructed in time on *B* after the initial preparation of |*ϕ*_*a*_〉 on *A*), it is very difficult to sort out Ω_*ab*_(*N*) because in this case, the evolution of the |*ϕ*_*b*_〉 population will only be almost-periodic in time^8^.

Measure (iii) is intermediate between (i) and (ii) because as demonstrated in this paper, *I*(*N*) is the long time average (low pass filtered) transduction of the |*ϕ*_*b*_〉 time evolution population amplitude normally tracked by (ii). Furthermore, (iii) is not a static characterization of the *A*–*N*–*B* spectrum like in protocol (i) which is searching for the |*ϕ*_*a*_〉 to |*ϕ*_*b*_〉 energy splitting among the *A*–*N*–*B* eigenstates.

For low *V*_*ab*_(*N*) and after the generalization of the Bardeen perturbation approach of tunneling^9^, it was long demonstrated that in the tunneling regime *I*(*N*) = *N*^2^*J* where *J* is the elementary tunneling current intensity passing through a single transfer line of the quantum bus^10^,^11^. This simple *N*^2^ power law was recently questioned because for some specific molecular quantum bus, *I*(*N*) was found to be lower than elementary *J*^12^ while in other experiments, it was proven to be valid^13^. To clarify the situation, a complete demonstration of the *I*(*N*) variations as a function of *N* is proposed in this paper. The exact quantum transduction function is introduced in a way to pass from the |*ϕ*_*b*_〉 time dependent population amplitude to the *T*(*E*_*f*_, *N*) electronic transparency of the *M*_*A*_-A-N-B-*M*_*B*_ tunneling junction (nano-pads Fermi energy *E*_*f*_). This transduction process is frequency limited explaining why for large *V*_*ab*_(*N*), the *N*^2^ power law was recently questioned. At low bias voltage and according to the Landauer formula, *I*(*N*) is proportional to *T*(*E*_*f*_, *N*). But *T*(*E*_*f*_, *N*) is necessary limited from above by quantum normalization. As a consequence, large *V*_*ab*_(*N*) values cannot be measured using (iii).

In section 2, the spectral analysis of the corresponding *A*–*N*–*B* quantum Hamiltonians and of the time dependent quantum evolution after preparing *A*–*N*–*B* in the non stationary initial state |*ϕ*_*a*_〉 are provided in a way to determine the *V*_*ab*_(*N*) variations as a function of *N* and of the bus control parameters. Two types of quantum bus are used for this demonstration, with one or two quantum states per line. In Section 3, the exact transformation between the |*ϕ*_*b*_〉 time dependent population amplitude and *T*(*E*_*f*_, *N*) is presented showing how this transformation is a quantum to classical low pass filter transduction between a quantum time dependent phenomenon and the tunneling junction conductance. In section 4, this transformation is used to determine the validity of (iii) i.e. when the *N*^2^ law is applicable and what is measured if not. In conclusion, the consequences of the limitations of the quantum transduction at work in a tunneling junction are discussed in the perspective of improving the contact conductance between a molecular wire and its metallic nanopads.

## Spectral analysis and time dependent Heisenberg-Rabi oscillations

To interconnect *A* and *B* with a quantum bus, two type of multipath quantum systems are considered in the following with 1-state and then 2-states per transfer line to be able to use analytical solutions to determine *V*_*ab*_(*N*). A number *N* of those lines are interacting in parallel, equally and independently with states |*ϕ*_*a*_〉 and |*ϕ*_*b*_〉. A quantum bus with *N* 1-state per line is the first member of a family having an odd number of states per line i.e. with always one eigenstate of the corresponding bus Hamiltonian located in the middle of its spectrum. A quantum bus with 2-states per line is the second member of a family having an even number of states per line i.e. having no state in the middle of its spectrum^14^. The first member of this second family is simply the direct through space coupling between *A* and *B*. For a quantum bus, having or not an eigenstate located in the middle of its electronic spectrum has profound consequences on the measurability of large *V*_*ab*_(*N*) values through this bus.

### *N* transfer lines in parallel with 1-state per line

**Figure 1:**
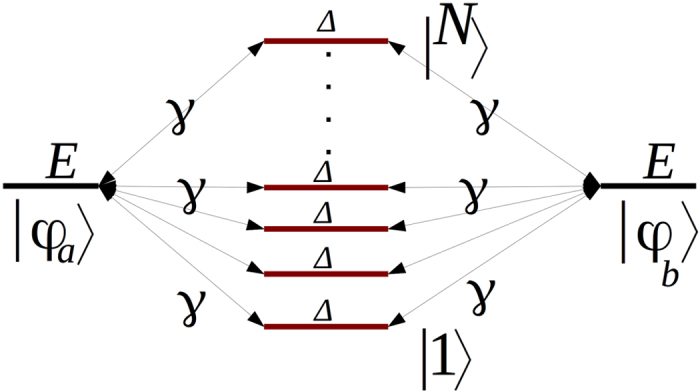
The quantum graph of an *N* 1-state per line bus interconnecting *A* and *B* with |*ϕ*_*a*_〉 for the electron on *A*, |*ϕ*_*b*_〉 the electron on *B* and |*j*〉 for the electron on the bus states. This determines the valence bond like canonical basis set of the system. The *N* parallel states have the same electronic energy Δ relative to |*ϕ*_*a*_〉 and |*ϕ*_*b*_〉 and are interacting with |*ϕ*_*a*_〉 and |*ϕ*_*b*_〉 via the electronic coupling *γ*.

Using the *A*–*N*–*B* canonical basis set |*ϕ*_*a*_〉, |*j*〉 (*j* = 1, *N*) and |*ϕ*_*b*_〉, Fig. 1 is presenting the complete N + 2 quantum states graph of a quantum bus with N 1-state per transfer line interacting with the emitter state |*ϕ*_*a*_〉 and the receiver state |*ϕ*_*b*_〉. Each 1-state line is *γ* interacting equally with |*ϕ*_*a*_〉 and |*ϕ*_*b*_〉 and there is a non zero energy difference Δ between the quantum bus states |*ϕ*_*a*_〉 and |*ϕ*_*b*_〉. This defines two quantum *γ*, Δ and one classical *N* control parameters for the *A*–*N*–*B* system.

The quantum properties of the Fig. 1 system have already been studied in detail for the purpose of engineering a bistable switch after playing with the electronic coupling of one transfer line^1^. We recall in this section the essential characteristics of this system not for switching but to focus on another aspect of its quantum controlability: the speed up of the electron transfer between *A* and *B* as a function of *N*. On its canonical basis set, the mono-electronic Hamiltonian of the Fig. 1 system is given by^1^:


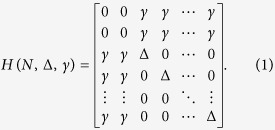


Its spectrum has *N* + 2 eigenvalues, *N* − 1 degenerated of value Δ and one *λ*_3_ = 0. The two remaining ones *λ*_1_ and *λ*_2_ are given by:


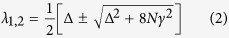


For 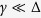, only two of those eigenvalues have their corresponding eigenvector very close to |*ϕ*_*a*_〉 and |*ϕ*_*b*_〉. In this case, the effective through bus coupling *V*_*ab*_(*N*) is simply 1/2 the energy splitting between *λ*_2_ and *λ*_3_ leading to 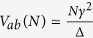 which is increasing linearly with *N*. For *γ* > Δ or for Δ = 0, the search for those two eigenvectors in the *H*(*N*, Δ, *γ*) spectrum is more difficult that in the previous case. For example for Δ = 0, the *λ*_3_ corresponding eigenvector has still the highest weight on |*ϕ*_*a*_〉 and |*ϕ*_*b*_〉. But at the same time, *λ*_1_ and *λ*_2_ have exactly the same weight. In the intermediate regime where *γ* and Δ are of the same order of magnitude, *λ*_2_ is still the second leading one and 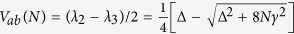 i.e. an 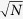 law for *V*_*ab*_(*N*).

Following protocol (ii), one way to determine *V*_*ab*_(*N*) for all the *γ* and Δ cases is to prepare the Fig. 1 system at *t* = 0 in the non-stationary state |*ϕ*_*a*_〉 to trigger a spontaneous response of the complete *A*–*N*–*B* system in time and to determine the effective Ω_*ab*_(*N*) oscillation frequency of the transfer process. As compared to the above spectral analysis for tracking *V*_*ab*_(*N*), the advantage of this preparation is that |*ϕ*_*a*_〉 is now specified and therefore by symmetry |*ϕ*_*b*_〉. After this preparation, the time response is given by the solution of the 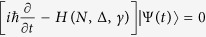 time dependent *Schrödinger Equation* leading after a projection on the canonical basis set used in Fig. 1 to the 3 coupled equations:


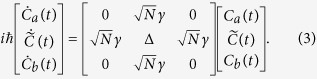


This system was obtained after calculating the |Ψ(*t*)〉, *C*_*a*_(*t*), *C*_*b*_(*t*), *C*_1_(*t*), …*C*_*N*_(*t*) coordinates on the canonical basis set, after taking into account the symmetry of the *A*–*N*–*B* system i.e. *C*_1_(*t*) = *C*_2_(*t*) = … = *C*_*N*_(*t*) = *C*(*t*) and finally after performing the transformation 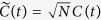 as implemented in ref. 2. After solving (3) analytically, the variation in time of the |*ϕ*_*b*_〉 population amplitude is given by:


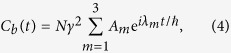


where 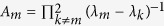 with *λ*_*i*_ for i = 1, 2, 3 the eigenvalues of 3. The population of the target state |*ϕ*_*b*_〉 is given by:


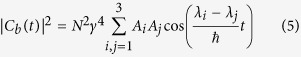


This almost periodic function leads to resonant and anti-resonant time dependent evolutions for well defined *γ* and Δ values. For Δ = 0, |*C*_*b*_(*t*)|^2^ is always periodic for all *N*. For Δ ≠ 0 such a resonant regime is reached only when *γ*Δ^−1^ takes the values^1^:


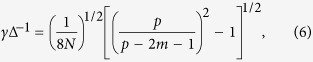


for integer *p* and *m*, and for 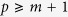 and *p* ≠ 2*m* + 1. As a consequence and whatever *γ*Δ^−1^, a 1-state per line bus always permits to reach |*ϕ*_*b*_〉 from |*ϕ*_*a*_〉 in time with no attenuation in average of the |*C*_*b*_(*t*)|^2^ maximum amplitude over time as a function of *N*.

Since there is one zero eigenvalue for the reduced Hamiltonian (3), *C*_*b*_(*t*) is the sum of two power 2 sinusoidals of frequency 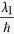 and 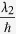. This is a generic property of a quantum bus with an odd number of states per line. Then, the Ω_*ab*_(*N*) effective oscillation frequency between |*ϕ*_*b*_〉 and |*ϕ*_*a*_〉 is given by the largest component in (5). For non zero *γ* and Δ, the largest component is *A*_3_*A*_1_ and the secular frequency is given by:


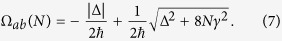


According to (7) and for 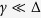, 
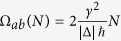
 is linearly dependent on *N* as already demonstrated in the spectral analysis above. For 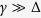, Ω_*ab*_(*N*) is following a 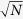 moderate increase with *N*. For resonant Δ = 0, 
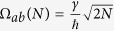
 because here the eigenvalues of the 2 eigenstates involved in the transfer process are 
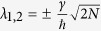
. Those 3 last cases were not accessible in the above spectral analysis and are leading to an effective *V*_*ab*_(*N*) proportional to 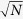.

### *N* transfer lines in parallel with 2-states per line

**Figure 2:**
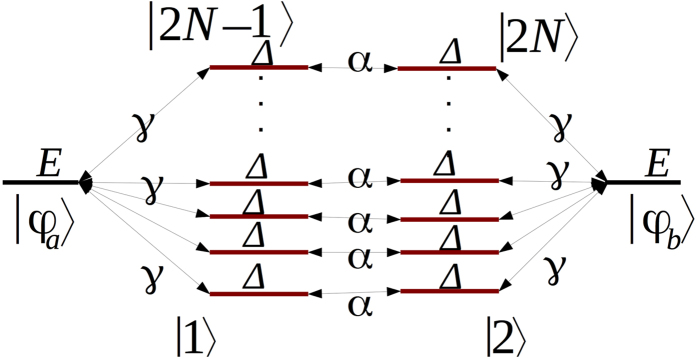
The quantum graph of the *N* 2-states per line bus interconnecting *A* and *B* with |*ϕ*_*a*_〉 for the electron on *A*, |*ϕ*_*b*_〉 the electron on *B* and the 2*N* |*j*〉 states for the electron on a given state on the bus. Those 2*N* states of the bus have the same electronic energy Δ relative to |*ϕ*_*a*_〉 and |*ϕ*_*b*_〉. They are interacting with |*ϕ*_*a*_〉 and |*ϕ*_*b*_〉 via the electronic coupling *γ. α* is the electronic coupling between 2 states along the same transfer line.

Using the *A*–*N*–*B* canonical basis set |*ϕ*_*a*_〉, |*j*〉 (*j* = 1, …, 2*N*) and |*ϕ*_*b*_〉, Fig. 2 is presenting the complete 2N + 2 quantum states graph of the second quantum bus considered in this work with N 2-state per transfer lines interacting with the emitter state |*ϕ*_*a*_〉 and the receiver state |*ϕ*_*b*_〉. Each 2-states line is *γ* interacting equally with |*ϕ*_*a*_〉 and |*ϕ*_*b*_〉 and there is also a non zero energy difference Δ between the quantum bus states and |*ϕ*_*a*_〉, |*ϕ*_*b*_〉. This defines three quantum *α*, *γ*, Δ and one classical *N* control parameters for this second *A*–*N*–*B* system.

On its canonical basis, the mono-electronic Hamiltonian of the Fig. (2) system reads:


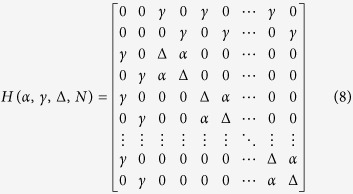


Its spectrum has 2*N* + 2 eigenvalues, *N* − 1 are degenerated of value Δ − *α*, *N* − 1 degenerated of value Δ + *α* and the 4 last ones are given by:


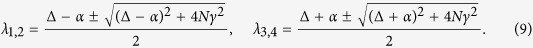


For 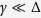, two cases are observed. For 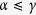, two of those eigenvalues, *λ*_2_ and *λ*_4_, have their corresponding eigenvector very close to |*ϕ*_*a*_〉 and |*ϕ*_*b*_〉. This leads to 
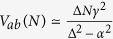
, which is increasing linearly with *N* as for a 1-state per line bus case. For *α* > *γ*, the two concerned eigenstates are now the ones with their respective eigenvalues *λ*_4_ and *λ*_1_ leading to 
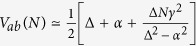
 also linearly depending on *N*. For 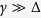 down to *γ* = Δ, two cases are also observed. For 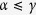, 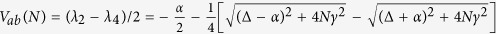. For *α* > *γ*, the two eigenvalues corresponding with their respective eigenvectors closed to |*ϕ*_*a*_〉 and |*ϕ*_*b*_〉 are now *λ*_1_ and *λ*_4_ leading to 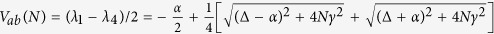 leading finally to an 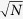 law for *V*_*ab*_(*N*). This is also obtained for Δ = 0 leading to 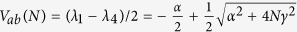. Finally, there are cases where this spectral analysis does not allow to determine the effective coupling *V*_*ab*_(*N*). For example, when *α* = *γ* = Δ, the *λ*_4_ eigenvector has still the highest weight on |*ϕ*_*a*_〉 and |*ϕ*_*a*_〉. But at the same time, *λ*_1_ and *λ*_2_ have exactly the same weight, which makes the selection of only two eigenstates difficult in this case.

Following protocol (ii), Ω_*ab*_(*N*) and therefore *V*_*ab*_(*N*) can be determined in all cases by preparing the Fig. 2 system at *t* = 0 in the non stationary state |*ϕ*_*a*_〉 triggering a spontaneous response of the complete *A*–*N*–*B* system in time. As compared to the spectral determination of *V*_*ab*_(*N*), the advantage of this preparation is here again that |*ϕ*_*a*_〉 is now specified and therefore |*ϕ*_*b*_〉 by symmetry. After this preparation, the time response is given by the solution of the 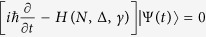 time dependent *Schrödinger Equation* leading after a projection on the canonical basis set used in Fig. 2 to the 4 coupled equations:


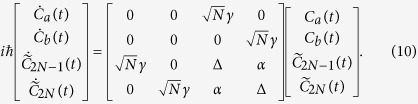


Following the section 2.1 approach, this system was obtained after calculating the |Ψ(*t*)〉. *C*_*a*_(*t*), *C*_*b*_(*t*), *C*_1_(*t*), …, *C*_2*N*_(*t*) coordinates on the canonical basis set, after taking into account the symmetry of the *A*–*N*–*B* system i.e. *C*_1_(*t*) = *C*_3_(*t*) = …*C*_2*N*−1_(*t*), *C*_2_(*t*) = *C*_4_(*t*) = …*C*_2*N*_(*t*) and finally after performing the transformation 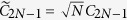 and 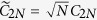. After solving (10) analytically, the variation in time of the |*ϕ*_*b*_〉 population amplitude is given for this quantum bus by:


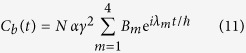


where 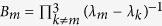. The population of the target state is then simply given by


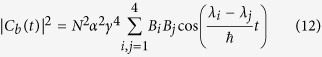


Contrary to the 1-state per line case, the maximum |*C*_*b*_(*t*)|^2^ population over time in not unity for all the *N* values. But as compared to (6), there is no analytical determination possible of the resonant and anti-resonant *α*, *γ* and Δ values as a function of *N*. We have not pushed further this analysis to concentrate on the dominant Heisenberg-Rabi oscillation frequency of the quantum oscillation process through this 2-states per line bus.

Since there is no zero eigenvalue for the reduced Hamiltonian (10) and since its spectrum is symmetric, *C*_*b*_(*t*) is the sum of 4 simple sinusoidal terms. This is a generic property of quantum bus with an even number of states per line. As a consequence, there are six different oscillation frequencies in (12): Ω_*ij*_ = (*λ*_*i*_ − *λ*_*j*_)/*ħ* for *i*, 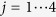 with *i* ≠ *j* weighted by *B*_*i*_*B*_*j*_.

For *α* < Δ, the largest coefficient in (12) is *B*_2_*B*_4_ with the corresponding Heisenberg-Rabi oscillation frequency Ω_*ab*_(*N*) = Ω_24_ given by:


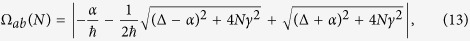


leading for 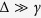 to 
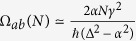
 which is linearly dependent on *N*. But for 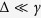 or Δ = *γ*, Ω_*ab*_(*N*) is following a 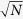 moderate increase with *N*.

For *α* > Δ, the largest coefficent in (12) is now *B*_1_*B*_4_ with the corresponding Heisenberg-Rabi oscillation frequency Ω_*ab*_(*N*) = Ω_14_ given by:


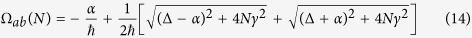


leading also for 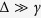 to 
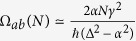
 which is linearly dependent on *N*. For 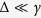 or Δ = *γ*, Ω_*ab*_(*N*) is again following a 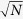 law. Notice that the variation of Ω_*ab*_(*N*) as a function of *α* is not a continuous function with an effective frequency jump for *α* = Δ. This explains the above change of the largest coefficient in (12) between *B*_2_*B*_4_ and *B*_1_*B*_4_.

For the resonant case Δ = 0, the largest coefficient in (12) is also *B*_1_*B*_4_ leading to the corresponding Ω_*ab*_(*N*):


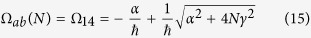


which is linearly dependant on *N* for 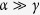 and is following a 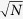 law for *α* ≤ *γ*.

Finally and in the very peculiar case *α* = *γ* = Δ, the two coefficients *B*_1_*B*_4_ and *B*_2_*B*_4_ in (12) are equal. This makes the analytical calculation of the corresponding Heisenberg-Rabi oscillation frequency very cumbersome and for 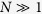, the Heisenberg-Rabi frequency becomes 
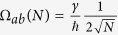
.

## Discussion

The above detail analysis was necessary to appreciate the richness of the time dependant quantum behaviour of 1-state and 2-states per line quantum bus. For 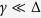 and for the 2 types of bus, Ω_*ab*_(*N*) and therefore *V*_*ab*_(*N*) is always increasing linearly with *N*. This is obtained for both the spectral (i) and the time dependent approach (ii).

When *γ* ≥ Δ or when Δ = 0, the spectral analysis (i) is not able to capture the richness of the quantum mixing between |*ϕ*_*a*_〉, |*ϕ*_*b*_〉 and all the other states of the canonical basis set. In this case, the interest of preparing a non stationary initial state like |*ϕ*_*a*_〉 is to ease for the determination of *V*_*ab*_(*N*) via Ω_*ab*_(*N*) when the control parameters are not permitting a clear spectral identification of the eigenstate participating the most in the construction of |*ϕ*_*a*_〉 and of |*ϕ*_*b*_〉 by symmetry. Starting from |*ϕ*_*a*_〉, the determination of the Heisenberg-Rabi secular oscillation frequency is a good way to pick up over time the two pertinent eigenstates. By practicing this protocol (ii) preparation, the Ω_*ab*_(*N*) variations with *N* are generally showing a 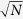 variation which is not the intuitive superposition law mentioned in the introduction.

## Measuring Ω_*ab*_(*N*) using a tunnel junction

Following now protocol (iii), the measurement of *V*_*ab*_(*N*) using Ω_*ab*_(*N*) requires that *A* and *B* interact electronically with two metallic nano-pads *M*_*A*_ and *M*_*B*_ respectively using states |*ϕ*_*a*_〉 and |*ϕ*_*b*_〉 as the two pointer states of the electron transfer process from *M*_*A*_ to *M*_*B*_ through *A*–*N*–*B*. With no bias voltage applied to the *M*_*A*_-A-N-B-*M*_*B*_ junction, *M*_*A*_ will sometime and randomly transfer one electron to A (or *M*_*B*_ to B). In this case, no potential different results between *M*_*A*_ and *M*_*B*_ and the required *A*–*N*–*B* elementary charging energy is coming from thermal fluctuations since *M*_*A*_ and *M*_*B*_ are necessarily in interactions with some thermal reservoirs, for example the surface supporting the *M*_*A*_-A-N-B-*M*_*B*_ junction^15^. When a low bias voltage difference *V* is applied between *M*_*A*_ and *M*_*B*_, a net current flows through the junction and its intensity *I*(*N*) is given by the Landauer formula^16^:


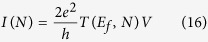


where 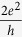 is the quantum of conductance.

Averaged in time, *I*(*N*) results from a large number of electrons transferred events per second occurring from *M*_*A*_ to *M*_*A*_ through *A*–*N*–*B*^15^. From *A* to *B* through the quantum bus, each individual electron transfer event is described by an Heisenberg-Rabi time dependent quantum oscillations as discussed in section 2. At low bias voltage, we model the quantum measurement at work on this process and performed by the *M*_*A*_-A-N-B-*M*_*B*_ junction by the transformation:


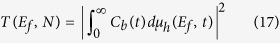


where *C*_*b*_(*t*) is the population amplitude of state |*ϕ*_*b*_〉 defined in section 2 for the two types of bus. With (17), the intrinsic quantum time evolution running in the junction is not eliminated but filtered and transduced to give rise to *T*(*E*_*f*_, *N*). For a low electronic coupling between |*ϕ*_*a*_〉 and |*ϕ*_*b*_〉 through the bus, different *μ*_*h*_(*E*_*f*_, *t*) transduction functions have already been proposed in the past and even a *μ*_*h*_(*E*, *t*) for large V. It is generally a time dependent damping exponential to avoid the divergence when calculating (17)^17^,^18^,^19^ or to reproduce the low pass filtering effect of a tunnel junction^20^. This is also what was anticipated by Lipmann and Schwinger^21^ to eliminate the fast time variations near and on the scattering center from their model of quantum scattering and to be able to work only with asymptotic states far away from this scattering center.

**Figure 3:**
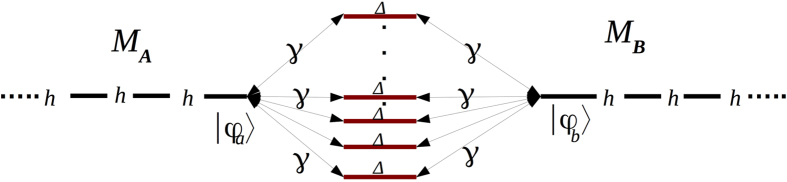
The schematic model of two semi-infinite quantum chains to model the *M*_*A*_ and *M*_*A*_ nano-pads interacting with the simple Fig. 1 *A*–*N*–*B* quantum system. *h* is the interstate coupling along those chains and Δ is the energy difference between those chains states and the central *A*–*N*–*B* system.

To determine *μ*_*h*_(*E*_*f*_, *t*), we have applied (17) to the Fig. 1 *A*–*N*–*B* system. Here, each line of this 1-state per line bus is now interacting with 2 semi-infinite chains to model the *M*_*A*_ and *M*_*B*_ nano pads, each with a single conduction band and a 4 h bandwidth as presented in Fig. 3. For simplicity, |*ϕ*_*a*_〉 and |*ϕ*_*b*_〉 are supposed to have the same energy than the on-site energy of an electron propagating site by site along the *M*_*A*_ or the *M*_*B*_ chains. *C*_*b*_(*t*) and *T*(*E*_*f*_, *N*) can be both calculated analytically with *C*_*b*_(*t*) given by (4) and *T*(*E*_*f*_, *N*) given in ref. 1:


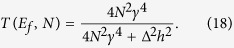


After some calculations to obtain (18) from (17) and after the introduction of (4) in (17), the *μ*_*h*_(*E*_*f*_, *t*) measurement function reads:


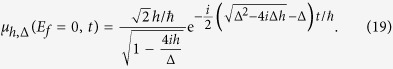


At *E*_*f*_, (19) can in fact be applied to any quantum system introduced in the tunnel junction if it is interacting with the nano-pads only via the pointer states |*ϕ*_*a*_〉 and |*ϕ*_*b*_〉. In particular when there are no quantum states in the junction, a small Ω through space electronic coupling can remain between *M*_*A*_ and *M*_*B*_. This is exactly the conditions used by J. Bardeen^9^ to get the low bias voltage V tunneling current intensity 
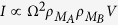
 through a simple *M*_*A*_–Ω–*M*_*B*_ tunneling junction where 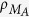 and 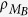 are the *M*_*A*_ and *M*_*B*_ electronic density of states. For the simple Fig. 3 *M*_*A*_ and *M*_*B*_ conducting chains 
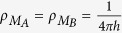
 leading to:


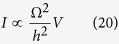


For this simple case, the corresponding *μ*_*h*_(*E*_*f*_, *t*) measurement function is given for *E*_*f*_ = 0 by:


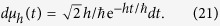


In this case and disconnecting now the two *M*_*A*_ and *M*_*B*_ measurement chains to return to the measurement protocol (ii), it remains a 2 states isolated quantum system |*ϕ*_*a*_〉 and |*ϕ*_*b*_〉 with a through space electronic coupling Ω between them. As described in section 2 and preparing this simple system in the non-stationary state |*ϕ*_*a*_〉, the time variations of the |*ϕ*_*b*_〉 population amplitude during the Heisenberg-Rabi oscillation process is simply *C*_*b*_(*t*) = sin Ω*t*/*ħ*. Then, using (20) and inserting this *C*_*b*_(*t*) in (17) leads to:


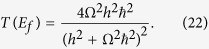


which is the exact *T*(*E*_*f*_) one can calculate analytically applying a simple scattering approach on a valence bond like canonical mono-electronic basis set^22^. Interestingly, (22) reduces to (20) for 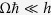 confirming that at low coupling and for this very simple *M*_*A*_–Ω–*M*_*B*_ quantum system *T*(*E*_*f*_) is proportional to the square of the *C*_*b*_(*t*) oscillation frequency^15^. This indicates that *μ*_*h*_(*E*_*f*_, *t*) is rather universal. Its extension for the complete energy range of the *M*_*A*_ and *M*_*B*_ measurement bandwidth is now under exploration.

As exemplified with (16) and also for the simple *M*_*A*_–Ω–*M*_*B*_ Bardeen tunnel junction, (17) with (19) is able to pick up at low coupling the secular oscillation frequency of *C*_*b*_(*t*) leading to *T*(*E*_*f*_, *N*) ∝ Ω_*ab*_(*N*)^2^. There is a limit for the functionning of this transduction because *T*(*E*_*f*_, *N*) is bond from above to unity and as demonstrated in section 2, *V*_*ab*_(*N*) and then Ω_*ab*_(*N*) are monotonically increasing with *N*. This limit manifests itself by the peculiar variation of *T*(*E*_*f*_, *N*) as a function of Ω_*ab*_(*N*) when Ω_*ab*_(*N*) is increasing so much that *T*(*E*_*f*_, *N*) is saturating to unity.

**Figure 4:**
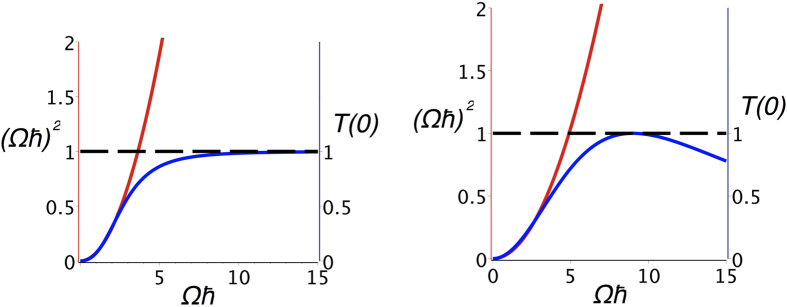
Illustration of the transduction process saturation at work in a tunneling junction. *C*_*b*_(*t*) in (17) is here simulated by sin(Ω*t*/*ħ*) for an odd number of state per line case (left) and by sin^2^(Ω*t*/*ħ*) for an even number of states per line (right). In both cases, the red curve is indicating the Ω^2^ variation expected at low coupling for *T*(*E*_*f*_). Due to the *T*(*E*_*f*_) normalisation to unity and also to the low pass filter character of the *μ*_*h*_(*E*_*f*_, *t*) transduction function in (17), *T*(*E*_*f*_) is either saturating (left) or even decaying (right) when Ω is increasing indicating the quantum limitation of this transduction process which can be tuned by changing the value of *h* in *μ*_*h*_(*E*_*h*_, *t*).

According to (4) and (14), *C*_*b*_(*t*) is a linear superposition of sinusoidal terms with different oscillation frequencies. To understand the functioning of (17) and since under its modulus, (17) is a linear transformation, one can choose for *C*_*b*_(*t*) simply a sin(Ω*t*/*ħ*) or a sin^2^(Ω*t*/*ħ*) depending respectively of the odd or even number of state in the bus lines. The unique property of (17) is that for *C*_*b*_(*t*) = sin(Ω*t*/*ħ*), *T*(*E*_*f*_) will decrease for large Ω after reaching *T*(*E*_*f*_) = 1 while for *C*_*b*_(*t*) = sin^2^(Ω*t*/*ħ*), *T*(*E*_*f*_) will simply saturate to unity for large Ω (Fig. 4). This behaviour is at the origin of the debate in the literature about the validity of the *I*(*N*) = *N*^2^. *J* superposition law refs 1 and 12.

The second property of (17) is its low pass filtering character on any *C*_*b*_(*t*) due to the *μ*_*h*_(*E*_*f*_, *t*) exponential time dependant term. As already noticed in ref. 20, this implies that the large *C*_*b*_(*t*) frequency components will not be capture in *T*(*E*_*f*_) because for Ω > *h*/*ħ*, *T*(*E*_*f*_) is first saturating to unity. Therefore, (17) permits the determination of the secular *C*_*b*_(*t*) oscillation frequency using in (17) a well tuned *μ*_*h*_(*E*_*f*_, *t*) function that is with a good selection of the electronic bandwidth of the *M*_*A*_ and *M*_*B*_ nano-electrodes around *E*_*f*_.

## The parallel quantum circuit law

Knowing the general properties of the linear transformation (17) to pass from *C*_*b*_(*t*) to its corresponding *T*(*E*_*f*_), we can now discuss how the richness of the time dependent quantum behaviours of 1-state and 2-states per line bus discussed in section 2 are preserved or not through the (17) transduction effect corresponding to protocol (iii). For a 1-state per line bus, starting from (4) and using (17), the *T*(*E*_*f*_, *N*) analytical expression is given by:


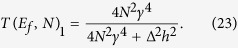


and for a 2-states per line bus using now (14) in (17):


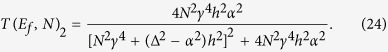


Both expressions can also be directly obtained using the Elastic Scattering Quantum Chemistry (ESQC) method starting from a mono-electronic Hamiltonian and calculating directly the corresponding scattering matrix^22^. By doing so, the time dependent Heisenberg-Rabi oscillations are not showing up explicitly since such scattering calculations are using asymptotic non perturbed by the central junction eigenstates of the *M*_*A*_ and *M*_*B*_ electrodes. This is not a problem for 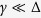 because in this case Ω_*ab*_(*N*) and therefore *V*_*ab*_(*N*) are not large enough to saturate (17) to unity assuming that *N* is small enough not to compensate for this small coupling through the bus. This becomes a problem for non tunneling regime or when the number *N* of lines in the bus is compensating for the initial low coupling through a single line. In this case, there is generally no more relation between *T*(*E*_*f*_, *N*) and the increasing Ω_*ab*_(*N*) as a function of *N*. In effect, for 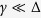 and for moderate *N* values, (23) and (24) are leading to a *N*^2^ variations for both *T*(*E*_*f*_, *N*)_1_ and *T*(*E*_*f*_, *N*)_2_ as a function of *N* up to the point where the increase in *N* is compensating for the initial small *γ* value. In this case, the *N*^2^ law is no more valid with a saturation of *T*(*E*_*f*_, *N*)_1_ whatever *N* and a decreasing of *T*(*E*_*f*_, *N*)_2_ for large *N* after its saturation to unity.

**Figure 5:**
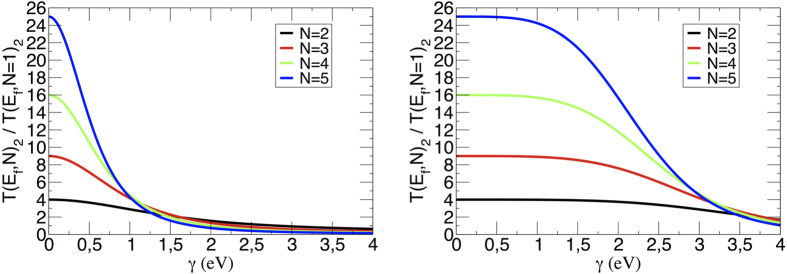
The ratio between *T*(*E*_*f*_, *N*)_2_ and *T*(*E*_*f*_, *N* = 1)_2_ for a bus with *N* 2-states transfer lines mounted in parallel for Δ = 0.0 eV (left) and Δ = 10.0 eV (right) with *h* = 4.0 eV and 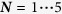 calculated at Fermi energy.

In Fig. (5), the range of the *N*^2^ law validity is presented by plotting the 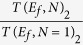 ratio for a 2-states per line bus. For small *γ* (Fig. 5 right panel), the *N*^2^ law is valid at least for *N* going from 1 to 5. But for large *γ*, this is no more the case. As discussed in section 3 and illustrated in Fig. 4, this is caused by the property of the transformation (17). Notice that this *N*^2^ law is valid for any odd and even number of states per line in the bus as soon as the increase in *N* is not compensating the 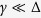 tunneling condition. Notice that for 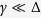, this *N*^2^ superposition law is a generalisation for the case of N quantum conductance connected in parallel of the 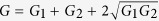 superposition law known in a tunnel regime for 2 quantum conductances *G*_1_ and *G*_2_ connected in parallel via a quantum node^11^ since for this peculiar *N* = 2 case, it comes *G* = 4*g* for *G*_1_ = *G*_2_ = *g*.

More interesting are now the cases where *γ* is closed or larger than Δ or when Δ = 0. Here, the value of *h* relative to Δ must also be considered because *h* is determining the energy range of the (17) transduction function. Furthermore and according to (23) and (24), *h* is playing the same role than *α*, *γ* and Δ in controlling this transduction. In this case and as discussed in section 2, there are many quantum control parameters values where Ω_*ab*_(*N*) is only proportional to 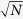.

The case raised up by C. Lambert and co-workers and underlined in the introduction is corresponding exactly to *N* = 2 for a 2-states per line bus with Δ = 0 and *α* = *γ* = *h*^12^. For this case, section 2.2 is giving 
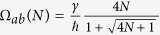
 which is following a 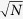 increase with *N*. But using now (24) for this C. Lambert case, 
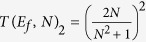
 leading for *N* = 2 to 
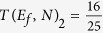
. There is here a notable decrease in electronic transparency in passing from *N* = 1 to *N* = 2 since for *N* = 1, *T*(*E*_*f*_, *N*)_2_ = 1. This clarifies the literature debate concerning the *N*^2^ power law. It turns out that the case raised up in ref. 12 is not a tunneling case. Already for *N* = 2 and since Δ = 0 and *α* = *γ* = *h*, *T*(*E*_*f*_, *N*)_2_ is already in its decaying regime for an *N* increase due to the properties of the transduction function (17).

To push further the discussion using (23) and (24), it is important to notice that for a 1-state per line bus, *N*^2^ is appearing both at the numerator and the denominator of (23). Therefore and after a *T*(*E*_*f*_, *N*)_1_ saturation to unity for large *N*, it is impossible to follow the richness of any quantum behaviour observed in section 2 using the transduction (17). This is for example the case of the 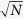 variations of Ω_*ab*_(*N*) for large *N*. For a 2-states per line bus and aside from the C. Lambert case Δ = 0, there are many interesting Heisenberg-Rabi time-dependent quantum behaviours which can be capture by (24) since now there is an *N*^4^ term in the *T*(*E*_*f*_, *N*)_2_ denominator and only an *N*^2^ in its numerator. In (24), the (Δ^2^ − *α*^2^) term is also playing a important role. For example for the case *α* = Δ = *h* = *γ* i.e. where according to section 2.2, 
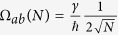
, the decreasing of Ω_*ab*_(*N*) with a *N* increase is well captured by (17) leading to 
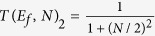
. This indicates how important is the *h* tuning to follow the Ω_*ab*_(*N*) variations with *N* i.e. to optimize the transduction process at work in a tunnel junction.

## Conclusions

We have started by analysing the quantum spectral properties of 1-state per line and 2-states per line bus with the objective to determine how the *V*_*ab*_(*N*) effective electronic coupling through such bus between an emitter and a receiver states varied as a function of the number *N* of lines connected in parallel to form this bus. For cases where it was spectrally difficult to determine *V*_*ab*_(*N*), we have re-enforced this analysis by triggering an Heisenberg-Rabi time dependent through bus quantum exchange process with an effective secular oscillation frequency Ω_*ab*_(*N*). For this purpose, we have prepared a specific initial non-stationary pointer state and used its symmetric target pointer state to determine Ω_*ab*_(*N*). This leads to two different Ω_*ab*_(*N*) (and therefore *V*_*ab*_(*N*)) regimes of variations as a function of *N*: a linear one following the intuitive superposition of electronic couplings and a 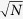 moderate increase as a function of *N*. In a way to substitute the initial pointer state preparation by electronically coupling the quantum bus with semi-infinite electrodes, we have discussed how the quantum transduction measurement process at work in such a tunneling junction can or not faithfully follow the variation with *N* of the through bus *V*_*ab*_(*N*) effective electronic coupling. Due to the normalisation to unity of the electronic transparency of any quantum bus and to the low pass filter like character of the transduction process at work in a tunnel junction, large *V*_*ab*_(*N*) increase due to an *N* increase cannot be detected by a tunneling junction. The *N*^2^ superposition law is preserved for Ω_*ab*_(*N*) (and therefore *V*_*ab*_(*N*)) for low coupling as soon as *N* is small enough not to compensate for this small through bus coupling per line. The limitations of the quantum transduction at work in a tunneling junction is also pointing out how the broadly used concept of electrical contact between a metallic nanopad and a molecular wire may be better described in term of a quantum transduction process. This is opening the way for a better optimisation of this transduction process at work in a tunneling junction for example by engineering the electronic band structure of the conducting nanopads in charge of this transduction to optimize the so-called contact conductance.

## Additional Information

**How to cite this article**: Namarvar, O. F. *et al.* Parallel Quantum Circuit in a Tunnel Junction. *Sci. Rep.* **6**, 30198; doi: 10.1038/srep30198 (2016).
